# Identification of a Novel Intracellular Interaction Domain Essential for Bves Function

**DOI:** 10.1371/journal.pone.0002261

**Published:** 2008-05-21

**Authors:** Michiya Kawaguchi, Hillary A. Hager, Aya Wada, Tatsuki Koyama, Min S. Chang, David M. Bader

**Affiliations:** 1 Stahlman Cardiovascular Research Laboratories, Cardiovascular Medicine, Department of Medicine, Vanderbilt University, Nashville, Tennessee, United States of America; 2 Department of Ophthalmology and Visual Sciences, Vanderbilt University, Nashville, Tennessee, United States of America; Illinois Institute of Technology, United States of America

## Abstract

While Blood vessel epicardial substance (Bves) confers adhesive properties, the molecular mechanism of regulating this activity is unknown. No predicted functional motifs in this highly conserved integral membrane protein, other than the transmembrane domain, have been identified. Here, we report for the first time that Bves interacts with itself through an intracellular interaction domain that is essential for its intercellular adhesion activity. Glutathion-S-transferase (GST) pull-down and SPOTs analyses mapped this domain to amino acids 268-274 in the intracellular C-terminus. Site-directed mutagenesis revealed that lysines 272 and 273 are essential for homodimerization and cell adhesion. Human corneal cells transfected with wild-type Bves trafficked the protein to the cell surface, assembled junction complexes and formed epithelial sheets. In contrast, cells expressing Bves mutated at these positions did not form continuous epithelial sheets or maintain junctional proteins such as ZO-1 and E-cadherin at the membrane. A dramatic reduction in transepithelial electrical resistance was also observed indicating a functional loss of tight junctions. Importantly, expression of mutated Bves in epithelial cells promoted the transformation of cells from an epithelial to a mesenchymal phenotype. This study is the first to demonstrate the essential nature of any domain within Bves for maintenance of epithelial phenotype and function.

## Introduction

Bves was discovered independently by Reese et al. [1] and Andree et al. [2] and is the prototypical member of the Popeye domain containing (*popdc*) gene family[2]. It is highly conserved and has been identified in a wide variety of vertebrate and invertebrates [1]–[5]. Both mRNA and protein of Bves are highly expressed in striated and smooth muscle and in various forms of epithelial cell types in the embryo and adult [1], [2], [5]–[11]. Biochemical analyses have determined that Bves is an integral membrane protein [12], [13], while localization studies have found Bves at the lateral cell membrane and within vesicles of the Golgi apparatus [12], [13]. Still, no molecular understanding of protein function is currently available.

Bves has the canonical structure of all predominant *popdc* gene products. This includes a short extracellular N-terminus with two invariant glycosylation sites, three transmembrane domains with two intervening loops and a long intracellular C-terminus [2], [9], [12]. While Bves has a highly conserved primary amino acid sequence among different species, there are no studies identifying any protein domain linked to any molecular or cellular function.

Phenotypic analyses of this gene family are only now emerging. Due to its subcellular localization and trafficking to points of cell-cell contact during epithelial sheet formation [9], we proposed that Bves might play a role in cell-cell adhesion. Transfection of Bves into normally non-adherent L-cells conferred adhesive activity [13] much like E-cadherin indicating that the transfected molecule confers adhesive properties 14, 15. Additionally, morpholino knockdown of Bves protein inhibited epithelial sheet formation and stability, and disrupted transepithelial electrical resistance (TER) [9]. While *popdc1*-null mice do not show an overt embryonic phenotype, presumably due to redundancy of expression with *popdc2* and *popdc3* genes, regeneration of skeletal muscle is delayed due to an inhibition of cell-cell adhesion/interaction [16]. Early inhibition of Bves function in *Drosophila* development results in disruption of pole cell migration [4], while gastrulation in *X. laevis* is severely restricted due to failure in epithelial morphogenesis [17]. Still, no reports have identified any functional domains within Bves or described the molecular basis of Bves function for adhesion or any other possible activities in tissue or organ morphogenesis.

Here for the first time, we report a Bves-Bves molecular interaction through its intracellular C-terminus that is essential for molecular regulation of cell-cell adhesion. This domain lies within the highly conserved Popeye region of the molecule, which heretofore has no ascribed function. Further we identify two amino acids in this sequence (K^272^ and K^273^) that are critical for homophilic binding. While transfection of wild type Bves promotes cell aggregation in L-cell assays, mutation or deletion of K^272^ and K^273^ abolishes this activity. Expression of these mutated transcripts dominantly interferes with normal Bves function in human corneal epithelial cells (HCE) resulting in loss of cell-cell adhesion, junction formation, TER and epithelial sheet integrity. Importantly, expression of mutated Bves leads to a change of cells from an epithelial to mesenchymal phenotype. This study is the first to identify a specific molecular mechanism by which Bves regulates cell-cell adhesion and to demonstrate that mutation of these sequences inhibits cellular functions attributed to this molecule.

## Results

### Bves intermolecular interaction through the intracellular C-terminus

**Figure 1:**
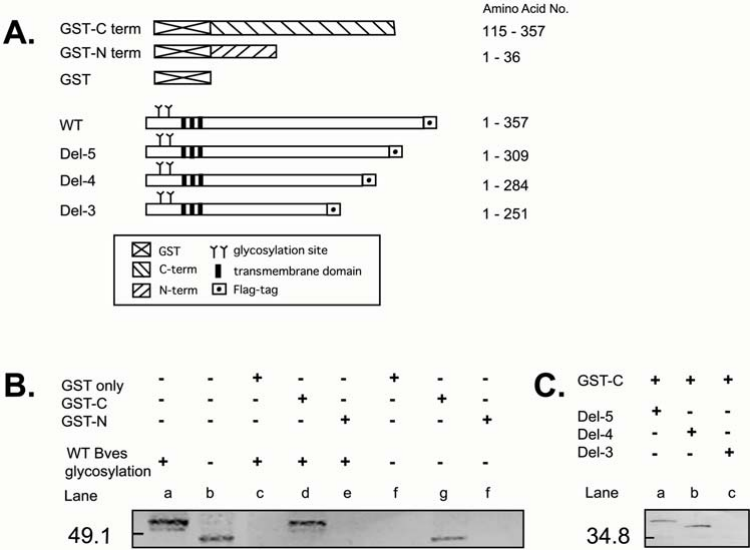
Bves constructs and GST pull-down assay. A, Diagram of GST-fusion proteins and deletion constructs. GST C- and N-terminal Bves proteins and serially deleted Flag-tagged Bves proteins. B, GST pull-down assay with C- or N-terminal Bves. Bacterial lysates of GST-Bves fusion proteins were reacted with COS-7 cell lysates transfected with Flag-tagged WT Bves and analyzed with Western blots using an anti-Flag antibody. The potential effects of glycosylation were also tested. Control band (WT Bves protein with or without glycosylation) are shown on lane a and b. Only GST C-terminal Bves reacted in this assay regardless of glycosylation state (lanes d and g) C, Serial deletion analysis of C-terminal interaction. GST C-terminal Bves was reacted with Del-5 Bves, Del-4 Bves and Del-3 Bves and processed for Western blotting to detect interactions. Del-5 Bves and Del-4 Bves (lanes a and b) interact with the GST C-terminal Bves while Del-3 Bves does not.

The molecular basis of Bves adhesive function is unknown [13]. To determine the molecular mechanisms that underlie this function, we explored whether Bves-Bves intermolecular interactions could be detected. We generated an array of wild type and truncated Bves constructs to identify possible Bves-Bves interaction domains (Figure 1A). In a first set of experiments, Flag-tagged Wild Type (WT) Bves harvested from COS-7 cells was reacted with GST N- or C-terminal Bves produced in E. coli. As seen in Figure 1B, GST C-terminal Bves readily precipitated WT Bves, while GST N-terminal Bves and GST alone did not. Elimination of the two N-terminal glycosylation sites had no effect on C-terminal interaction (Figure 1B, lane g). These results do not exclude the possibility of N-terminal interactions but demonstrate direct association between molecules through the C-terminus of Bves. To further define sequences in the C-terminus responsible for this activity, a series of C-terminal truncations (shown in Figure 1A) were reacted in similar manner. Deletion of C-terminal up to aa 284 (Del-4 Bves) had no effect on precipitation efficiency (Figure 1C) but elimination of the next 33 amino acids (Del-3 Bves) completely abolished C-terminal interactions. This construct, Del-3 Bves, was used further as a non-interacting control in subsequent studies. These data are the first to identify Bves-Bves homodimerization.

### Amino acids K^272^ and K^273^ are critical for Bves-Bves interaction

**Figure 2:**
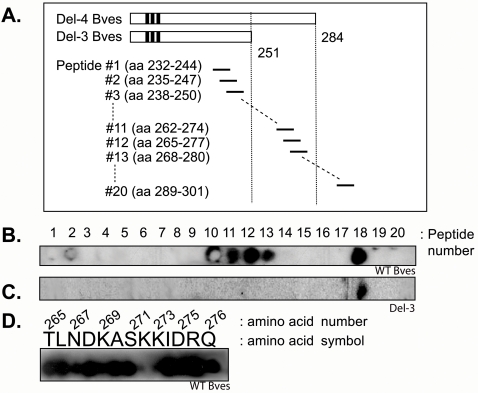
SPOTs protein mapping. A, Diagram of SPOTs blot (synthesized peptides). Twenty 13-mer peptides (SPOTs) were synthesized from the sequence of C-terminal Bves (amino acids 232-301) and fixed on a cellulose membrane for reaction with WT Bves or Del-3 Bves. B, Spots binding assay with WT Bves. A binding with WT Bves is clearly detected with peptides 11-13. Peptide 10 is likely a false positive, since the signal appears circumferential to the spot of protein. An additional unpredicted reaction is detected with peptide 18. C, Spots binding assay with Del-3 Bves. No binding of Del-3 Bves is detected with peptides 11-13 while peptide 18 is reactive suggesting a non-specific or interaction domain-independent association. D, Alanine substitution analysis. Numbers over the amino acid symbols are the amino acid number of WT Bves. Peptide 12, that showed positive binding at panel B was substituted at each amino acid position with alanine and incubated with WT Bves cell lysate. Substitution of lysines at positions aa 272 and aa 273 with alanine abolished binding with WT Bves protein.

To further define the domain responsible for Bves-Bves intracellular interaction, a solid phase SPOTs methodology was employed. 13-mer peptides were synthesized from position 232 to 301 from the intracellular tail of Bves encompassing the putative interaction domain (neighboring peptides have 10 amino acid overlap, Figure 2A). These peptides were incubated with either WT Bves or Del-3 Bves that is missing the putative interaction domain. As seen in Figure 2B, WT Bves binds peptides 11-13 as predicted from the liquid phase precipitation analysis as these peptides lie within the 33 aa interaction domain. The apparent reactivity around, not within SPOTs 2 and 10 are spurious and do not appear in other reactions with the membrane. An additional, unpredicted interaction is detected in peptide 18. When the same blot is reacted with Del-3 Bves (Figure 2C), no reaction with peptides 11-13 was observed while reactivity with peptide 18 remained. WT Bves binding with peptide 18 may be a non-specific reaction or represent additional Bves-Bves interaction independent of aa 268-274.

Next, to determine whether specific amino acids are required for binding activity, alanine substitution of individual amino acids across this core element was conducted and the resulting peptides were reacted with WT Bves. Figure 2D shows that lysine to alanine substitution at positions 272 and 273 eliminates Bves binding to the core element peptide. Interestingly, peptides 11 to 13, which are reactive in SPOTs analysis, are the only targets that contain both K^272^ and K^273^. These data indicate that aa 268-274 are required for at least one Bves-Bves interaction and that K^272^ and K^273^ are critical for this function.

### The intracellular interaction domain is essential in Bves-mediated cell-cell adhesion

**Figure 3:**
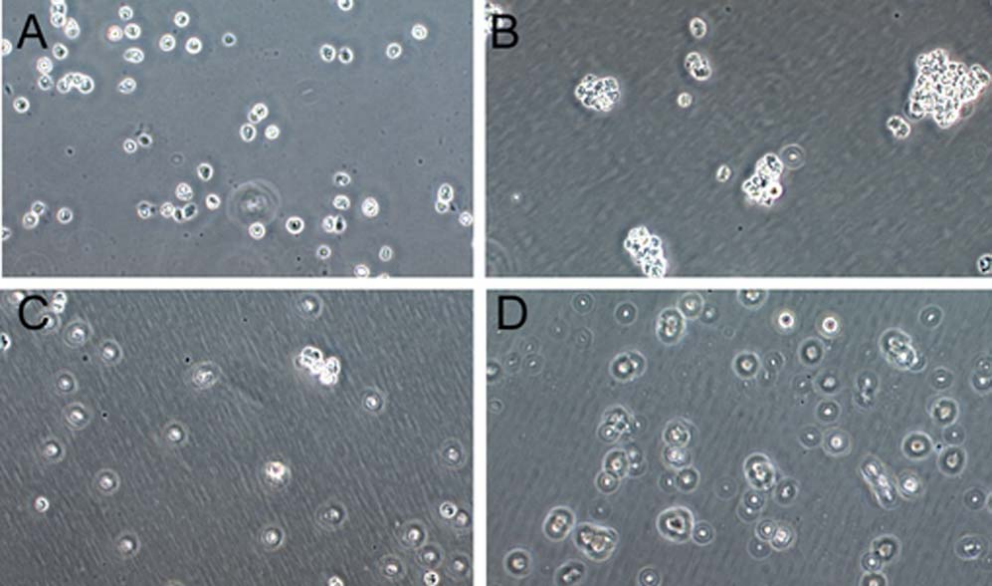
L-cell aggregation assay. Aggregation assays with various cell lines were performed using a standard hanging-drop method. A, Parental L-cells. B, L-cells transfected with WT Bves, C, KK-Mut Bves and D, KK-Del Bves. (Scale Bar 50 µm)

L-cells have been used to demonstrate adherent properties of transfected gene products (Thorson et al, 2000; Wada et al, 2001). As seen in Figure 3, non-transfected cells (3A) are non-adherent while transfection of WT Bves (3B) induces cell clustering as previously seen in Wada et al. We next explored whether the specific amino acids determined to be essential for C-terminal molecular interaction were critical for Bves adhesive function at the cellular level. L-cells were transfected with the two mutated forms of Bves, KK-Mutant (KK-Mut) Bves (Figure 3C) and KK-Deletion (KK-Del) Bves (Figure 3D). Both of these transfected cell lines failed to aggregate above levels seen in non-transfected parental cells (compare 3A, C and D). Taken together, these data demonstrate that mutation of the newly-identified Bves-Bves intracellular interaction domain abolishes the adhesive function.

### Expression of mutated Bves inhibits formation and stability of epithelial sheets

**Figure 4:**
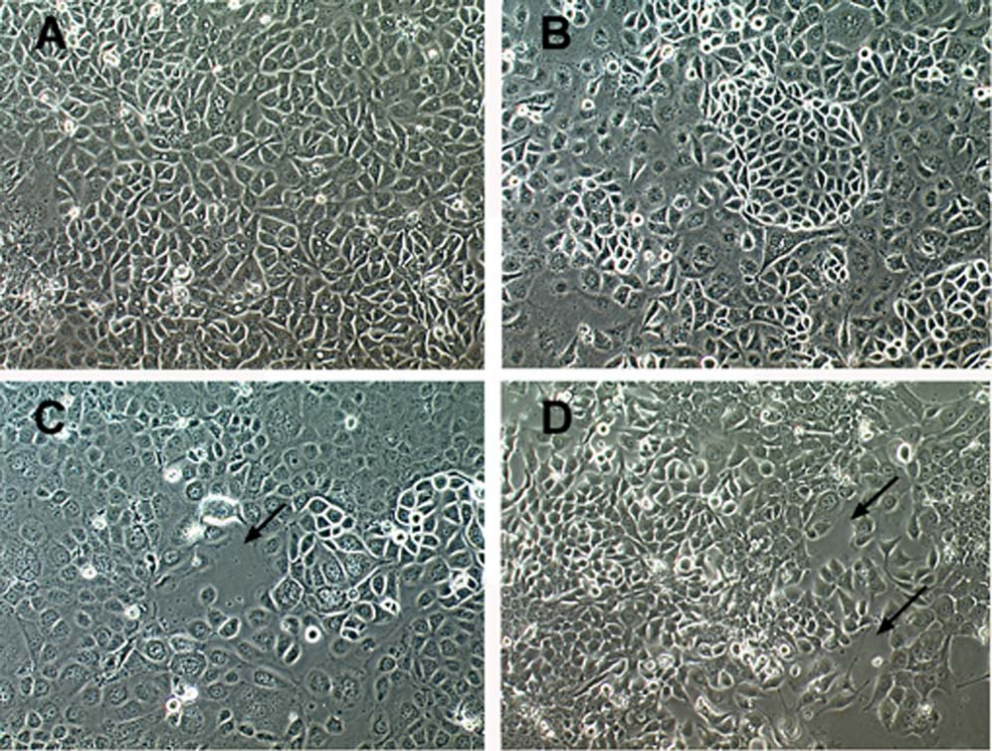
Phase contrast of transfected human corneal epithelial (HCE) cells. Non-transfected parental cells (panel A) form confluent epithelial sheets, as do HCE cells transfected with WT Bves (panel B). However, in WT Bves transfected cells, areas of tight adherence are readily observed (arrow head). In HCE cells transfected with mutant Bves, a contiguous monolayer is rarely formed. KK-Mut Bves transfected cells are more rounded and moderate gaps are viewed (panel C, arrows), while KK-Del transfected cells display distinct gaps.

We next determined whether expression of mutated Bves molecules would disrupt native adhesive properties in epithelia using a human corneal epithelial cell line (HCE). Multiple stable HCE cell lines expressing WT Bves, KK-Mut Bves and KK-Del Bves were isolated and produced consistent results. As seen in Figure 4A in phase microscopy, parental HCE lines form confluent epithelial monolayers when seeded at a high density. HCE cells transfected with WT Bves (Figure 4B) also develop confluent monolayers, however, areas exhibiting very densely packed epithelium are observed (arrowhead). Cells expressing a mutated form of Bves, KK-Mut Bves, do not form completely confluent monolayers, even when seeded at high densities. Irregular gaps in the epithelium are observed (arrow Figure 4C), and cells appear rounded. A more pronounced phenotype is seen in cells expressing KK-Del Bves (Figure 4D). This cell line never forms a confluent epithelium. Several gaps between cells are observed (arrows) as these cells are unable to form a monolayer.

**Figure 5 pone-0002261-g005:**
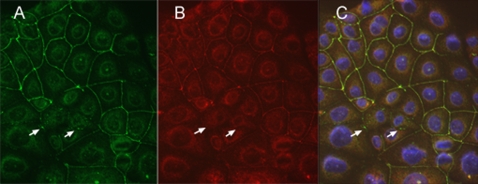
Distribution of transfected WT Bves in HCE cells. A stable cell line expressing WT Bves was grown to confluence and examined for Bves expression and distribution. Panel A demonstrates distribution of anti-Bves staining while B displays anti-Flag staining. Note that most staining overlaps (Panel C, merge) but small areas are observed that are positive for anti-Bves and negative for anti-Flag (arrows in A–C).

Using HCE cells as a model, we have shown that transfected WT Bves is properly trafficked to and maintained at the cell membrane (merge in Figure 5). It should be noted a minor but consistent population of endogenous Bves molecules, identified positive staining with anti-Bves antibodies (Figure 5A) and negative staining with anti-Flag (arrows, Figure 5B).

**Figure 6 pone-0002261-g006:**
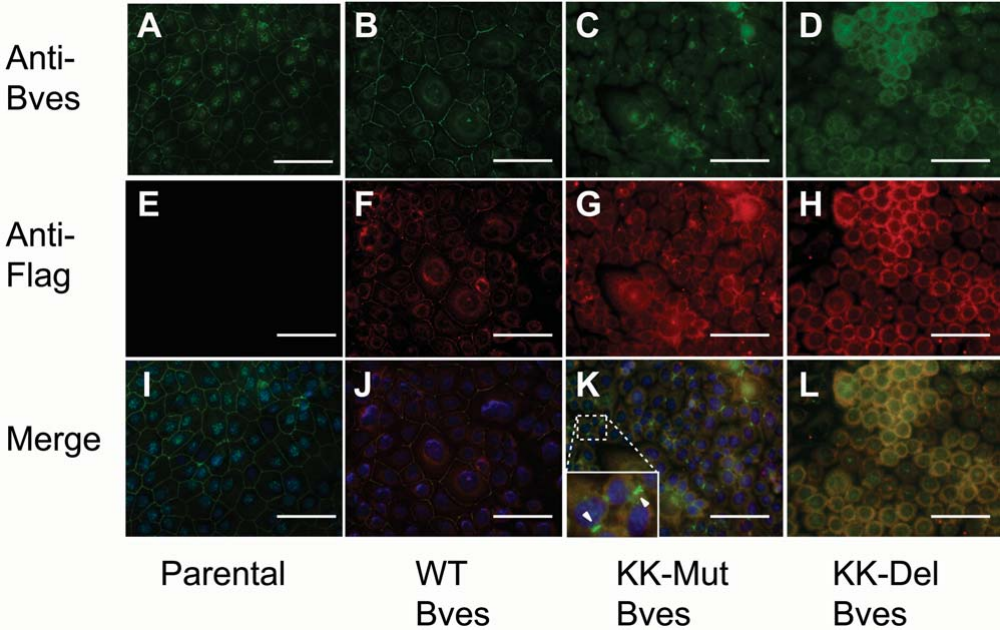
Detection of endogenous and transfected Bves in human corneal epithelial cells (HCE). Anti-Bves staining (top panels, A–D), anti-Flag staining (middle panels, E–H) and merge (bottom panels, I–L) are shown. A, Endogenous Bves is detected at the parental cell membrane in confluent monolayers. E, Parental cells do not react with anti-Flag. B, Endogenous Bves is expressed in WT Bves transfected cells. F, Transfected WT Flag-tagged Bves traffics to the cell membrane J, where it co-localizes with anti-Bves staining, which detects endogenous protein. C, G, K, Transfection of KK-Mut Bves and D, H, L, KK-Del Bves show a general loss of membrane staining for both endogenous and transfected protein. Inset K, importantly, sporadic green staining at the membrane is seen in merged images with transfected KK-Mut Bves suggesting localization of endogenous but not transfected protein. (Scale Bar 100 µm)

To detect transfected protein and endogenous Bves, cells were stained with anti-Flag and anti-Bves antibodies. Transfected WT Bves is detected at the cell membrane in these lines, and in areas of confluence, staining of both antibodies is observed around the entirety of cells. Intracellular staining of transfected and endogenous protein were also observed (Figures 5 and 6, panel J). Expression of either KK-Mut Bves or KK-Del Bves disrupts the pattern of anti-Bves staining in cells at high density. Flag-tagged protein is rarely observed at the cell surface and accumulates intracellularly (Figure 6G, H). It should be noted that all images were exposed and electronically processed identically. Thus, the reduced intensity of staining in Figure 6F and J can be compared to the greater signal observed in Figures 6G and H. In addition, the expression of a KK-Mut Bves inhibits trafficking and/or accumulation of endogenous Bves at the cell surface (Figure 6K). Still, in isolated areas, anti-Bves staining without overlapping anti-Flag staining was observed at the cell surface between adjacent cells indicating the presence of endogenous protein in the absence of transfected Bves. Anti-Flag immunoreactivity around the entire cell was rarely observed. In KK-Del Bves transfected cells distinct circumferential anti-Bves staining was also difficult to detect. These cells appeared smaller and had a significant intracellular accumulation of transfected proteins.

**Figure 7 pone-0002261-g007:**
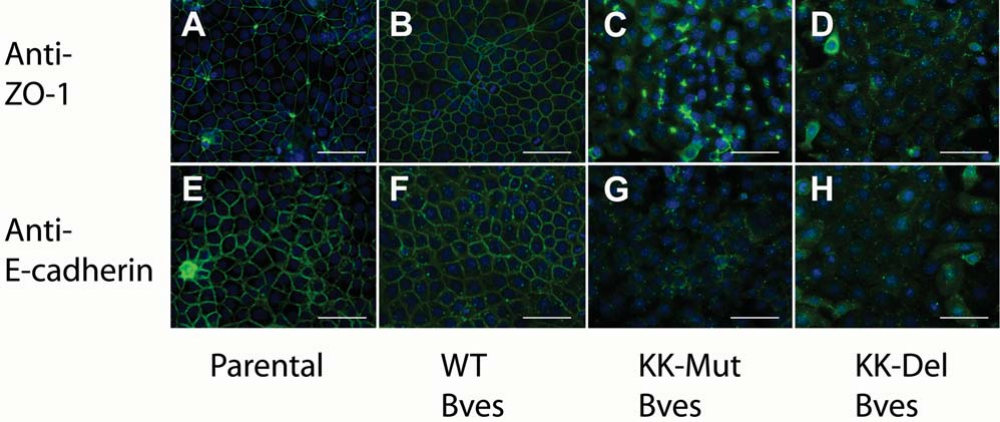
Expression of ZO-1 and E-cadherin in HCE cells. A, E, Parental HCE cells B, F, HCE cell lines transfected with WT Bves, C, G, KK-Mut Bves, and D, H, KK-Del Bves were stained with ZO-1 in panels A through D and E-cadherin in panels E through H. Note the loss of peripheral ZO-1 and E-cadherin staining in KK-Mut Bves and KK-Del Bves cells. (Scale Bar 100 µm)

To determine whether mutations of Bves at positions 272 and 273 disrupt junctional adhesive complexes, cultures were assayed for the distribution of known junctional proteins. As seen in Figure 7, ZO-1 and E-cadherin, components of the tight and adherens junctions, respectively, are drastically redistributed in cell lines expressing exogenously mutated forms of Bves. ZO-1 staining is seen in small patches but not with that of the altered forms of the protein. Circumferential staining of ZO-1 and E-cadherin were rarely if ever detected in cells expressing KK-Mut Bves or KK-Del Bves.

**Figure 8:**
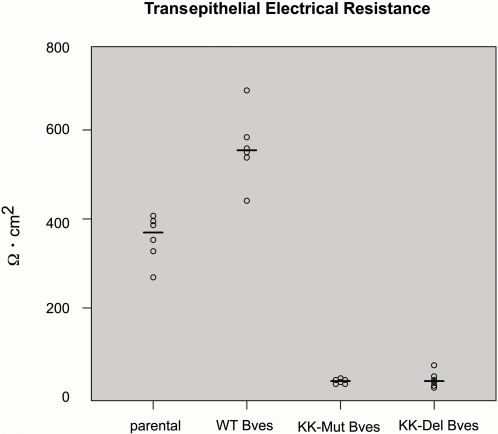
Measuring of transepithelial electrical resistance in HCE cells. Parental cells and cells expressing transfected WT Bves, KK-Mut Bves and KK-Del Bves were grown to confluence and assayed for transepithelial electrical resistance (TER) using EVOM volt-ohm-meter. Expression of transfected WT Bves increases TER. Conversely, cells expressing KK-Mut Bves or KK-Del Bves have TER values approaching background. The horizontal line indicates the median.

TER is a standard measurement of epithelial function and tight junction integrity. In order to determine the effect of mutation or substitution of K^272^ and K^273^ on tight junction activity, HCE cell lines stably transfected with WT Bves, KK-Mut Bves and KK-Del Bves were grown to confluence and assayed for TER (Figure 8). Transfected cultures were compared to the non-transfected parental cell line. A resistance of ∼350Ω cm^2^ was observed with non-transfected controls while transfection with WT Bves produced elevated resistance. When we measured TER in HCE cell lines expressing KK-Mut Bves or KK-Del Bves, values were dramatically reduced to near background levels. TER for KK-Mut Bves [p-value<0.0001, CI (0.07, 0.14)] and KK-Del Bves [p-value<0.0001, CI (0.07, 0.15)] are significantly reduced compared to the control parental group, while TER for WT Bves is significantly greater than those of the control parental group [p-value = 0.006, CI (1.08, 2.28)]. Taken together, these data suggest the expression of Bves with mutation in the putative intracellular interaction domain produced marked disruption of epithelial structure and function.

### Expression of mutated Bves leads to changes in epithelial cell phenotype

**Figure 9:**
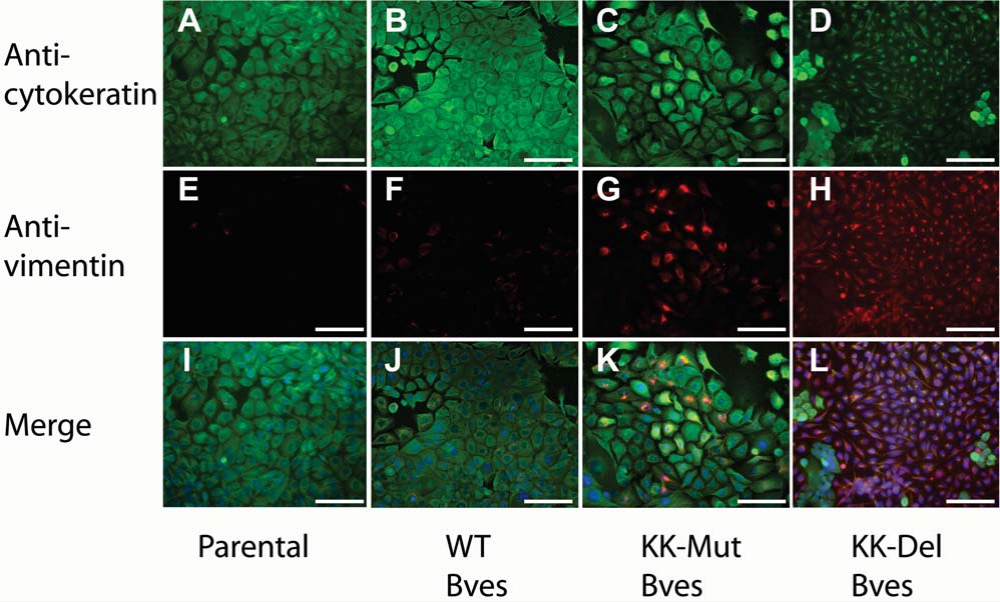
Immunostaining with anti-cytokeratin and anti-vimentin. Immunostaining with anti-cytokeratin (panels A through D), anti-vimentin (panels E through H) and merge (panels I through L) are given. A, E, I, Parental cells show high levels of cytokeratin and little vimentin staining. Transfection with WT Bves (B, F, J) shows similar staining patterns as parental cells. In contrast, cell lines expressing KK-Mut Bves (C, G, K) have a subpopulation of vimentin-positive cells and KK-Del Bves expressing cells (D, H, L) show nearly complete conversion to a vimentin-positive phenotype. (Scale Bar 100 µm)

Alteration of cell-cell adhesion/interaction can lead to changes in cell phenotypes, most notably through processes involving epithelial-mesenchymal transition [18]. Control parental and WT Bves-transfected cells expressed cytokeratin (Figure 9) and other markers of the epithelial phenotype with no expression of mesenchymal phenotype. In contrast, cells stably transfected with KK-Mut Bves expressed and accumulated vimentin, a marker of mesenchymal phenotype [19]. The number of cells with this phenotype was constant but low (Figure 9G). In contrast, a high percentage of KK-Del Bves expressing cells exhibited a mesenchymal phenotype even under culture conditions that favor maintenance of the HCE epithelial phenotype (Figure 9H). These data suggest that mutation of the Bves-Bves intracellular interaction domain leads to changes in cells consistent with a mesenchymal phenotype.

## Discussion

Bves is a highly conserved transmembrane protein with cell-cell adhesion function that is expressed in a variety of epithelia and muscle from flies to humans. Previous studies suggest that the function of the *popdc* gene family of which Bves is a prototypical member has great implications for development and disease. Still, the molecular mechanisms underlying this function are completely unknown. Additionally, no protein motif or domain has been identified to account for any activity of this gene family. Here for the first time, we demonstrate Bves-Bves interaction and identify a functional domain in the Bves molecule that regulates this process. Further, we show that mutation or deletion of specific amino acids within this domain abolishes the cell-cell adhesion mediated by the molecule and leads to predictable changes in cell phenotype. These novel studies identifying Bves-Bves interaction and its regulation have larger implications for the understanding of Bves function in development and disease.

### Identification of an intracellular interaction domain in Bves

While members of the *popdc* family are highly conserved, neither Bves nor any other family member harbors a predicted protein motif that would account for its cell-cell adhesive function [1], [2], [5]. However, the presence of an intracellular Bves-Bves interaction domain responsible for membrane clustering of the molecule had been previously suggested by Professor Thomas Brand [20]. Here, our truncation analysis of the C-terminus demarcates a region (aa 252-284) that mediates Bves-Bves intermolecular interaction and is sufficient for at least one homophilic binding event (Figures 1 and 2). It should be noted that the present study does not preclude the presence of additional Bves-Bves C-terminal interactions. Within this newly identified domain, lysines at positions 272 and 273 are critical for molecular interaction and are conserved in Bves protein sequences in mouse, human and chicken (NCBI). The identification of an intracellular domain that is essential for intercellular adhesive activity is not unprecedented, as an intracellular juxtamembrane domain in E-cadherin is essential for its intercellular adhesion function [21]–[23]. As no previous studies have ascribed function to any motif within Bves, identification of this intracellular domain is critical for an understanding of the function of this molecule.

### Mutation of Bves disrupts cell-cell adhesion

Expression of mutated Bves does not promote cell clustering in L cells in contrast to expression of WT Bves controls (Figure 3). Also, expression of mutant constructs greatly inhibits adhesion properties in normally adherent HCE cells (Figure 4–8). These assay systems provide strong corroborating data that suggest a functional significance for this intracellular interaction domain in cell-cell adhesion. We propose that over-expression of KK-Mut Bves or KK-Del Bves acts in a dominant-negative or interfering fashion in adherent cells that normally express Bves.

Additionally, immunofluorescence analysis of HCE cells expressing transfected WT Bves detects the molecule at the cell surface along with components of the adherens and tight junctions (Figure 7) and expression of WT Bves increases TER in transfected cells (Figure 8). In contrast, over-expression of either mutant form of Bves inhibits a function of the endogenous Bves protein as epithelial sheet integrity is compromised. It is interesting to note that only small puncta of endogenous Bves are observed at the cell surface (Figure 6K). Additionally, surface staining of adhesion molecules such as ZO-1 is punctate and also greatly diminished in cells expressing mutated Bves (Figure 7). These results indicate that: 1) the KK-containing domain is important for proper trafficking and/or stability of Bves since KK-mutated Bves disturbs membrane localization of endogenous Bves, and/or 2) the KK-containing domain is involved in regulating the membrane localization of other adhesion molecules. Therefore it is plausible that inhibition of Bves-Bves intracellular interaction in turn disrupts the generation and/or maintenance of cell-cell adhesion. Bves is one of the first molecules to traffic to points of cell-cell contact and it interacts with ZO-1 a known mediator of cell-cell adhesion [9], [13]. Disruption of either one or both of these functions could inhibit the assembly or stability of forming junctions. This may lead to the loss of adhesion protein localization at the membrane and the observed drop in TER in epithelial cells expressing mutated Bves. These data support the hypothesis that Bves is a critical regulator of cell-cell interaction and clearly delineates essential intra-cellular interaction in the process. From these new data, we postulate that the intracellular interaction domain is critical for clustering of Bves molecules and that this aggregation is important for intercellular interaction functions of Bves and/or association with other protein components of cell-cell junctions.

### Inhibition of Bves function leads to changes in cell phenotypes

A key finding in the present study is that cells expressing mutated Bves exhibit an altered phenotype. Many, but not all, epithelial cells expressing this molecule take on varying morphologies and express vimentin, a marker of the mesenchymal phenotype [19]. These HCE cells, which normally do not exhibit mesenchymal behavior, take on this fate with altered Bves function and the lack of membrane localization and/or stability of the protein. This finding mimics the situation observed for Bves during coronary artery differentiation. There we have observed that, adherent epithelial cells of the developing epicardium express Bves at the lateral cell surface [13]. When individual cells undergo epithelial-mesenchymal transition, Bves is removed from the cell surface along with other adhesion molecules and cells assume a mesenchymal phenotype. It is possible that expression of mutated Bves inhibits cell-cell adhesion resulting in the production of mesenchyme not unlike that observed during those processes observed in vivo. Thus, it is possible that the regulation of cell-cell adhesion is controlled in part through inter-molecular association governed by the Bves intracellular interaction domain.

## Materials and Methods

### Bves constructs

Specific regions of the Bves molecule were cloned using Polymerase Chain Reaction (PCR) strategies for biochemical and cellular analysis. Primers are listed in Table 1. N-terminal (amino acid (aa) 1-36) or C-terminal (aa 115-357) regions of Bves were cloned into the EcoRI and XhoI site of pGEX-5X-1 (Amersham, Piscateway, NJ) for bacterial expression and the Sal I and Not I site of pCIneo (Promega, Madison, WI) for eukaryotic expression. Wild type (WT) Bves and a C-terminal deletion series [Del-5 Bves (aa 1-309), Del-4 Bves (aa 1-284) and Del-3 Bves (aa 1-251)] were Flag-tagged (YYKDDDDK) on their C-termini and inserted into the Sal I and Not I site of pCIneo. Alanine substitution and deletion of K^272^ and K^273^ of Flag-tagged Bves were produced by a sequential PCR method using primers listed in Table 1 and cloned into pCIneo. Sequences of all constructs were confirmed in the DNA sequencing core at Vanderbilt University.

**Table 1:** Sequences of primers used to generate Bves constructs

Primer Name	Direction	Sequence (5′→3′)	T an (°C)
5′ N-term	S	TTGACAGAATTCATGGACACTACGGCAATCAGC	57
3′ N-term	AS	CAGTATCTCGAGCTCCTTCCAGTTTTCACATGC	57
5′ C-term	S	TTGACAGAATTCAGACCGATCAAGATAGAGAAAGAGC	57
3′ C-term	AS	CAGTATCTCGAGATGACAGATTCGTTCAAGGCAGCCGCTG	57
5′ SalI cBves	S	AGAGCTAGCGTCGACTTCAAGATGGACACTACGGCA	57
3′ Not I/Flag WT	AS	TACATATGCGGCCGCCTACTTGTCATCGTCGTCCTTGTAGTCAGGCAGCCGCTGCAGCTC	57
3′ Not I/Flag Del-5	AS	TACATATGCGGCCGCCTACTTGTCATCGTCGTCCTTGTAGTCCCCGCGAAGAAACATCTGCAA	57
3′ Not I/Flag Del-4	AS	TACATATGCGGCCGCCTACTTGTCATCGTCGTCCTTGTAGTCGAGCTGTGAGCAAAGACTTGG	57
3′ Not I/Flag Del-3	AS	TGTACTTACATATGCGGCCGCCTACTTGTCATCGTCGTCCTTGTAGTCAATGAGATACTTAAAGATCTC	57
5′ Mut/Del	S	ATCTTGTTTTCCACGTGGCAA	55
3′ Mut/Del	AS	GAACCTGAAACATAAAATGAATG	55
5′ KK-Mut	S	CCTTAAATGACAAGGCCTCA**GCGGC**GATTGATCGGCAGCCAAGTCT	55
3′ KK-Mut	AS	AGACTTGGCTGCCGATCAATC**GCCGC**TGAGGCCTTGTCATTTAAGG	55
5′ KK-Del	S	CCTTAAATGACAAGGCCTCA-ATTGATCGGCAGCCAAGTCT	55
3′ KK-Del	AS	AGACTTGGCTGCCGATCAAT-TGAGGCCTTGTCATTTAAGG	55

Note: Bold and underline characters show the mutation sites and hyphen shows the deletion site

S, Sense ; AS, Antisense ; T an, annealing temperature

### Cells, transfection and production of stable cell lines

COS-7 cells (ATCC, Manassas, VA) and L-cell (CLL 1.3, ATCC) were cultured in Doulbecco's Modification of Eagle's Medium (DMEM, Mediatech, Herndon, VA) with 4mM L-glutamine, 1.5 g/l sodium bicarbonate, 4.5 g/l glucose and 1.0 mM sodium pyruvate supplemented with 10% fetal bovine serum (FBS, Atlanta biological, Lawrenceville, GA) and 10 µg/ml Penicillin-Streptomycin solution (Mediatech) Human corneal epithelial cells (HCE) were originally obtained from Dr. K. Araki-Sasaki (Osaka, Japan), maintained as previously described [24], and were grown in Defined Keratinocyte-SFM with growth supplement (Invitrogen, Grand Island, NY). L-cells were transfected with WT Bves, C-terminal Bves, N-terminal Bves, KK-Mut Bves and KK-Del Bves. Two µg of each construct were used for transfection with the FuGENE6 transfection reagent (Roche, Indianapolis, IN). After 72 hours, positive clones were selected in growth medium containing G418 (0.4 mg/ml) and resistant cells were maintained in medium with G418 (0.2 mg/ml). HCE cells were also transfected with WT Bves, KK-Mut Bves and KK-Del Bves as described above. Stably transfected cell lines were obtained by using medium with G418 (0.02 mg/ml). Production of Bves protein was confirmed by immunochemical staining with anti-Bves (B846, [13]) and anti-Flag (M2, Sigma, St. Louis MO) antibodies.

### GST pull-down assay

GST N-terminal, GST C-terminal Bves or GST proteins were prepared using standard methods [9] and mixed with WT Bves produced in COS-7 cells and incubated overnight at 4°C. Replicate WT Bves transfected COS-7 cells were incubated with tunicamycin (2 mg/ml) to produce WT Bves protein devoid of glycosylation products. Reactions were then applied to glutathione beads and rocked at 4°C for four hours. Beads were washed three times with Phosphate Buffered Saline (PBS) and the protein was eluted with SDS sample buffer. Eluted proteins were analyzed by SDS-PAGE and Western blot using anti-Flag antibody [1]. The same experiments were repeated with a series of C-terminal deletion constructs to identify the precise region required for Bves-Bves homophilic interaction. Dilutions of antibodies were: primary antibodies (anti-Flag, M2, Sigma 1∶1000), secondary antibody (anti-mouse IgG alkaline phosphatase (AP) -conjugated, Sigma 1∶10,000). Blots were developed in NBT/BCIP (Roche, Indianapolis, IN) in AP buffer.

### SPOTs Protein Mapping

To more precisely identify the Bves-Bves interaction region in the C-terminus, a SPOTs blot membrane was generated by SIGMA-GENOSIS (Woodland, TX). Peptides were designed from the sequence of the C-terminus of Bves after the third hydrophobic membrane-spanning region. Synthesized 13-mer peptides (starting from aa 232) were covalently fixed to a cellulose membrane with ten overlapping amino acids between neighboring SPOTs (Figure 2A). The 20 total SPOTs contain the putative intracellular interaction domain between Del-4 Bves and Del- 3 Bves (aa 252-284). These SPOTs were assayed for binding with WT Bves and Del-3 Bves. Both proteins were prepared from COS-7 cell transfection and were incubated with anti-Flag antibody at 4°C overnight to form a protein/antibody complex. The membrane was incubated in blocking solution (2% skim milk in TBST) for two hours at room temperature and then incubated with the protein/antibody complex for three hours at room temperature. After extensive washing in TBST, the membrane was incubated with secondary antibody (anti-mouse IgG horseradish peroxidase (HRP) conjugated, Sigma). Interactions were detected by chemiluminescence (ECL plus, Amersham UK Ltd., Buckinghamshire, England). The membrane was regenerated using manufacturer's recommendation.

### Alanine Substitution analysis

Identification of amino acid(s) critical for Bves-Bves interaction was performed on a second SPOTs membrane. On this SPOTs blot, each single amino acid of Peptide 12: TLNDKASKKIDRQ was individually substituted with L-alanine beginning with N-terminal amino acid and progressing to the C-terminal of the peptide; synthesized 13-mer peptides were fixed on a cellulose membrane. Blocking reaction, washing and development with WT Bves were identical to the protocol described above.

### L-cell aggregation assay

Cellular adhesion activity of non-transfected control and stable L-cell lines transfected with Bves constructs (WT Bves, KK-Mut Bves, and KK-Del Bves) was compared in standard hanging drop suspension cultures [14], [15]. Images were acquired using an inverted image microscope (Olympus IX70), an object lens (LCPlan FI 20X/0.40 Ph1), a camera (OPTRONICS MagnaFire-Model S60800) and software (MagnaFire 2.1A) at room temperature.

### Immunofluorescence assay

Immunofluorescent analysis of protein expression and distribution was similar to previously published studies [9], [13], [25]. HCE cells transfected with WT Bves, KK-Mut Bves and KK-Del Bves or non-transfected parental cells were seeded on a four well-chamber slide (Lab-Tek II, Nalge Nunc, Naperville, IL) and immunostained using following antibodies. Primary antibodies were: anti-Bves (B846, [13], 1∶200), anti-Flag (M2, Sigma, 1∶150), anti-ZO-1 (Zymed, South San Francisco CA, 1∶200), anti-E-cadherin (Sigma, 1∶200), anti-cytokeratin (DAKO, Carpinteria CA, 1∶200), and anti-vimentin (AMF17b, Developmental Hybridoma Bank, Iowa City IA, 1∶200). Anti-rabbit IgG conjugated with Alexa 488 (Molecular Probe, Eugene OR, 1∶3000) and anti-mouse IgG conjugated with Cy3 (Jackson ImmunoResearch Lab, West Grove PA, 1∶3000) were used as secondary antibodies. DAPI (Molecular Probes) was used at 1∶3000 to stain nuclei. Images were acquired using a fluorescence microscope (Olympus AX70 TRF), an object lens (Olympus UPlan APO 40X/0.85), a camera (OPTRONICS MagnaFire-Model S60800) and software (MagnaFire 2.1A) at room temperature.

### Transepithelial electrical resistance (TER)

To examine tight junction activity in confluent cultures, the TER was measured. Non-transfected and transfected HCE cells were used in this study. Cells (2×10^4^ cells/cm^2^) were seeded on a six well transwell chamber (cell culture insert, 0.4 µm pore, Falcon/BD lab ware, Franklin Lakes, NJ) and cultured for two weeks. TER was measured using an Epithelial volt-ohm-meter (EVOM) (World Precision Instruments, Sarasota, FL). TER was calculated as follows: TER = (Reading of EVOM - reading of blank)×surface area of the membrane (4.2 cm^2^). The data are log-transformed to stabilize variances. Analysis of variance was applied to test for overall difference, followed by Dunnett's method to compare the three experimental groups to the parental group while controlling the family-wise type I error rate at 5%. Simultaneous 95% confidence intervals of the group mean ratios were obtained using Dunnett's method. The reported p-values and confidence intervals are adjusted for multiple comparisons [26].
